# The associations between TMAO-related metabolites and blood lipids and the potential impact of rosuvastatin therapy

**DOI:** 10.1186/s12944-022-01673-3

**Published:** 2022-07-21

**Authors:** Xiaowei Xiong, Jian Zhou, Qiang Fu, Xiaowei Xu, Shaobin Wei, Shenghua Yang, Buxing Chen

**Affiliations:** 1 Department of Cardiology and Macrovascular Disease, Beijing Tiantan Hospital, Capital Medical University, Beijing, China; 2 Department of Cardiovascular Medicine, Shanghai East Hospital, School of Medicine, Tongji University, Shanghai, China; 3 Department of Cardiology, Beijing University of Chinese Medicine Third Affiliated Hospital, No. 51, Xiaoguan Street, Andingmenwai, Chaoyang District, Beijing, 100029 China

**Keywords:** Trimethylamine N-oxide, Atherosclerotic cardiovascular disease, Rosuvastatin, Blood lipids

## Abstract

**Background:**

Trimethylamine N-oxide (TMAO)-related metabolites are associated with the pathogenesis of atherosclerotic cardiovascular disease (ASCVD) and are known to disrupt lipid metabolism. The aims of this study were to evaluate the associations between TMAO-related metabolites and blood lipids and determine how lowering the lipid profile via rosuvastatin therapy influences TMAO-related metabolites.

**Methods:**

A total of 112 patients with suspected ASCVD were enrolled in this study. The levels of plasma TMAO-related metabolites, including TMAO, choline, carnitine, betaine, and γ-butyrobetaine (GBB), were analyzed by stable isotope dilution liquid chromatography-tandem mass spectrometry (LC/MS/MS) before and after rosuvastatin therapy in all patients. Statistical methods were used to detect the associations between TMAO-related metabolites and blood lipids and determine how rosuvastatin therapy alters the levels of these metabolites.

**Results:**

A significant positive correlation was found between TMAO and triglycerides (TG) (*r* = 0.303, *P* < 0.05). Furthermore, significant negative correlations were found between TMAO and high-density lipoprotein cholesterol (HDL-c) and between betaine and low-density lipoprotein cholesterol (LDL-c) (*r* = − 0.405 and − 0.308, respectively, both *P* < 0.01). Compared to baseline, significantly lower TMAO levels and higher carnitine, betaine and GBB levels were observed after rosuvastatin therapy, while the lipids decreased significantly (*P* < 0.05). The significant correlation between TMAO and TG or between betaine and LDL-c disappeared after rosuvastatin therapy (*r* = 0.050 and − 0.172, respectively, both *P* > 0.05). However, a significantly positive association between carnitine and TC and a negative association between carnitine and LDL-c or between betaine and TG were found after adjustment for sex, age, body mass index (BMI) and lipids (*P* < 0.05).

**Conclusions:**

This study suggests that TMAO-related metabolites are significantly associated with blood lipids, although some of them are changed postrosuvastatin therapy. Lower TMAO and higher TMAO precursors were observed after rosuvastatin therapy compared to baseline. This study indicates that elevated TMAO precursors after rosuvastatin therapy and their potential impact on ASCVD should be considered in the clinic.

**Supplementary Information:**

The online version contains supplementary material available at 10.1186/s12944-022-01673-3.

## Introduction

Trimethylamine N-oxide (TMAO) is an oxidative product of trimethylamine (TMA), which is generated from choline, choline-containing substances, L-carnitine, γ-butyrobetaine (GBB) and betaine by the gut microbiota [1–3]. TMAO and its related precursors mentioned above are collectively termed TMAO-related metabolites. Many studies have found that TMAO-related metabolites are associated with the formation and development of atherosclerotic cardiovascular disease (ASCVD) [1–4], and elevated TMAO-related metabolite levels are associated with a heavy atherosclerotic burden, poor prognosis of ASCVD and a high risk of major adverse cardiovascular events (MACEs) [5–8]. Atherosclerosis is a type of chronic metabolic disease that is characterized by chronic inflammation and lipid metabolism disorders [9]. The associations between TMAO-related metabolites and inflammation have been studied and demonstrated in cultured cells, mouse models, and even clinical trials [10–13]. Moreover, some studies also found that the compositions of the gut microbiota are associated with variations in blood lipids [14, 15], and TMAO-related metabolites, such as TMAO, carnitine and GBB, could disrupt cholesterol metabolism in animal models [2, 4, 16]. Although a previous study found that plasma betaine was negatively associated with lipids, such as low-density lipoprotein cholesterol (LDL-c) and triglycerides (TG) [17], there is still little information about the relationships between TMAO-related metabolites and blood lipids. In addition, as an effective lipid-lowering drug, rosuvastatin was found to influence gut microbiota composition and the function of genes related to the metabolism of TMAO-related metabolites [18, 19]. However, information about its effect on TMAO-related metabolites is still ambiguous. Therefore, the aims of this study were to investigate the associations between TMAO-related metabolites and blood lipids and examine the potential impact of rosuvastatin therapy on these metabolites and associations.

## Methods

### Study population

**Fig. 1:**
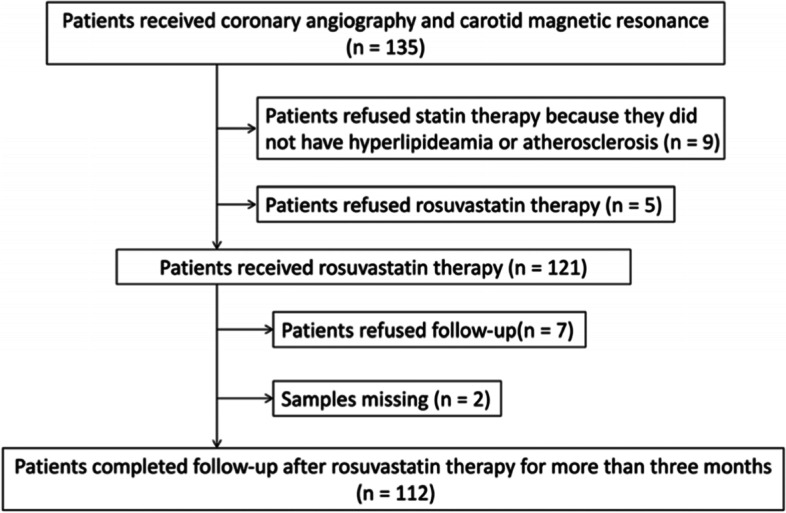
Flow chart

This is a prospective study based on a registered clinical trial at http://clinicaltrials.gov/ (No.02305862). Patients with suspected ASCVD who were willing to receive coronary angiography and carotid magnetic resonance imaging (MRI) were consecutively recruited from Beijing Tiantan Hospital between January 2013 and December 2016. The demographic characteristics, personal history and diagnosis were based on personal statements, clinical records, current drug usages, and auxiliary examinations in the hospital. The exclusion criteria were age < 18 years, stent implantation, uncontrolled hypertension, acute myocardial infarction, malignant tumor, heart failure, active infections, severe liver or kidney dysfunction, stroke, diabetes mellitus (DM), familial hyperlipidemia, and antibiotic usage. A total of 135 patients were recruited in this study, including 35 patients who received irregular statin therapy for less than 1 month and had a clearance period of at least 2 weeks. A total of 121 patients received rosuvastatin therapy because of hyperlipidemia, ASCVD, and/or other proper purposes. All patients were initially administered 20 mg rosuvastatin per night, and 4 of these patients were adjusted to 10 mg because of impaired liver function during the follow-up time. No patients suspended usage during the follow-up. All patients were given a recommended light diet before sampling and after discharge and monitored half-monthly at the outpatient department or by telephone interviews. Finally, 112 patients with complete follow-up data after 3 months of follow-up constituted the study cohort. The study flowchart is displayed in Fig. 1, and the baseline characteristics of the cohort are presented in Supplementary Table S1. This study abided by the principles of the Declaration of Helsinki and was approved by the Ethics Committee of Beijing Tiantan Hospital (Approval No: KY2014–020-02). All patients in this study signed informed consent forms. The data can be obtained from the corresponding author after the permission of the Ethics Committee of Beijing Tiantan Hospital.

### Laboratory testing

Fasting venous blood samples were collected using EDTA-containing pyrogen-free tubes after admission and immediately centrifuged at 4 °C and 3000 rpm for 10 min to obtain plasma samples. Then, the plasma samples were stored at − 80 °C until analyses. The TMAO, choline, carnitine, betaine and GBB levels were measured by stable isotope dilution liquid chromatography-tandem mass spectrometry (LC/MS/MS) according to previous methods [20]. Briefly, plasma samples (20 μl) were mixed with 80 μl of 5 μM internal standards of TMAO-trimethyl-d9 (d9-TMAO), choline-trimethyl-d9 (d9-choline), carnitine-trimethyl-d9 (d9-carnitine), betaine-trimethyl-d9-methylene-d2 (d11-betaine) and GBB-trimethyl-d9 (d9-GBB) in methanol. They were detected in MRM MS mode: d9-TMAO at *m/z* 85.0 → 65.9 amu, d9-choline at *m/z* 113.2 → 68.9 amu, d9-carnitine at *m/z* 162.0 → 102.8 amu, d11-betaine at *m/z* 118.0 → 59.0 amu and d9- GBB at *m/z* 146.1 → 87 amu. Different concentrations of TMAO, carnitine, choline, betaine and GBB standards and a fixed amount of internal standards were added to 4% bovine serum albumin to generate the calibration curves for the quantification of blood TMAO-related metabolites. The standard curve coefficient of determination (R^2^) reached 0.999, and the accuracy across a span of different concentrations all reached 95%. After protein precipitation, the supernatant was recovered after centrifugation at 20000 g at 4 °C for 10 minutes. Then, 70 μL of the supernatant was absorbed into vials for loading, and the injection volume by the autosampler was 3 μl. The detections were performed on an API 5500Q-TRAP mass spectrometer (AB SCIEX, Massachusetts, USA). Analytes were monitored in positive MRM MS mode by using the following characteristic precursor-production ion transitions: TMAO at *m/z* 75.7 → 59.0 amu, choline at *m/z* 104.0 → 59.8 amu, carnitine at *m/z* 162.0 → 85.0 amu, betaine at *m/z* 118 → 57.7 amu and GBB at m/z 155.1 → 87.0 amu. All stable iso-labeled internal standards used in this study were purchased from Cambridge Isotope Laboratories, Inc. (Andover, MA, USA). Lipid parameters, including TG, total cholesterol (TC), high-density lipoprotein cholesterol (HDL-c), LDL-c, apolipoprotein A1 (ApoA1) and apolipoprotein B (ApoB), were measured by a biochemistry autoanalyzer (Beckman Coulter DxC 800, California, USA) in the clinical laboratory center of Beijing Tiantan Hospital. Hyperlipidemia was defined as TC ≥ 6.22 mmol/L, LDL-c ≥ 4.14 mmol/L, TG ≥ 2.26 mmol/L or HDL-C < 1.04 mmol/L, which was based on the criteria recommended by the U.S. Adult Treatment Panel III.

### Statistical analyses

Categorical variables were compared by the chi square test and expressed as frequencies (n) and percentages (%). Normality of continuous variables was evaluated using a one-sample Shapiro–Wilk test. Student’s t test was performed to compare the differences between groups, or the Mann–Whitney test was used when variables were not normally distributed. A paired test was used to compare the differences in lipids and TMAO-related metabolites before and after rosuvastatin therapy. All data are expressed as the mean ± standard deviation (SD). Pearson analyses were performed to evaluate the correlations between TMAO-related metabolites and lipids, and Spearman analyses were performed when variables were not normally distributed. Partial correlation analyses were performed to explore the correlations between TMAO-related metabolites and lipids by adjusting for sex, age, body mass index (BMI) and estimated glomerular filtration rate (eGFR). Multivariate analyses were performed using a linear regression model to identify the associations between TMAO-related metabolites and lipids and investigate the effect of rosuvastatin therapy on TMAO-related metabolites. The variables included in the multivariate analyses were sex, age, BMI, eGFR, TG, TC, LDL-c, HDL-c, ApoA1 and ApoB. The statistical analyses were performed using SPSS version 17.0 (SPSS Inc., Chicago, IL). A *P* value < 0.05 was considered statistically significant.

## Results

### Baseline characteristics

In this study, female patients with suspected ASCVD were older (64.57 ± 6.94 vs. 60.34 ± 9.77 years, *P* < 0.05) and had lower rates of smoking (12.8% vs. 75.4%, *P* < 0.05) and drinking (2.1% vs. 41.5%, *P* < 0.05) than male patients (Supplementary Table S2), and these results are consistent with epidemiological characteristics. All patients were divided into low-TMAO and high-TMAO groups according to the median TMAO level (3.92 μM), and their baseline characteristics are shown in Table 1. Compared with patients in the low-TMAO level group, patients in the high-TMAO level group had older age, higher BMI, lower eGFR and HDL-c levels, and higher TG, choline and carnitine levels (*P* < 0.05).

**Table 1:** Baseline characteristics of the study cohort

Variables	TMAO ≤3.92 μM (*n* = 56)	TMAO > 3.92 μM (*n* = 56)	*P*
Male, n (%)	36 (64.3)	29 (51.8)	0.180
Age, years	60.18 ± 8.51	64.05 ± 8.96	0.021
BMI, kg/m^2^	25.53 ± 3.15	26.91 ± 3.73	0.036
Smoke, n (%)	28 (50.0)	27 (48.2)	0.850
Alcohol, n (%)	16 (28.6)	12 (21.4)	0.383
Hypertension, n (%)	43 (76.8)	42 (75.0)	0.825
Hyperlipidemia, n (%)	27 (48.2)	31 (55.4)	0.449
CHD, n (%)	45 (84.4)	49 (87.5)	0.303
eGFR, mL/min/1.73^m2^	101.01 ± 7.77	96.48 ± 7.69	0.002
TG, mmol/L	1.69 ± 0.68	2.19 ± 1.15	0.006
TC, mmol/L	4.27 ± 0.95	4.32 ± 1.03	0.795
HDL-c, mmol/L	1.32 ± 0.42	1.09 ± 0.32	0.002
LDL-c, mmol/L	2.64 ± 0.75	2.57 ± 0.74	0.589
ApoA1, mmol/L	1.27 ± 0.19	1.26 ± 0.18	0.753
ApoB, mmol/L	1.11 ± 0.33	1.09 ± 0.37	0.835
TMAO, μM	2.83 ± 1.34	8.43 ± 4.85	< 0.001
Choline, μM	12.50 ± 2.81	14.01 ± 3.09	0.008
Carnitine, μM	75.21 ± 15.69	82.58 ± 15.44	0.014
Betaine, μM	39.26 ± 10.90	38.06 ± 10.09	0.548
GBB, μM	0.09 ± 0.02	0.10 ± 0.02	0.059

Data are expressed as the mean ± standard deviation or n (%)

*TMAO* Trimethylamine N-oxide, *BMI* Body mass index, *eGFR* Estimated glomerular filtration rate, *CHD* Coronary heart disease, *TG* Triglycerides, *TC* Total cholesterol, *HDL-c* High-density lipoprotein cholesterol, *LDL-c* Low-density lipoprotein cholesterol, *ApoA1* Apolipoprotein A1, *ApoB* Apolipoprotein B, *GBB* γ-butyrobetaine

### Associations between TMAO-related metabolites and lipids before rosuvastatin therapy

First, we found that TMAO was positively correlated with patient age and BMI and negatively correlated with eGFR (*r* = 0.265, 0.247 and − 0.275, respectively, all *P* < 0.05), and betaine was negatively correlated with BMI (*r* = − 0.285, *P* < 0.05) (Supplementary Table S3 and Fig. S1). Second, the associations among the TMAO-related metabolites were significant except for the association between betaine and GBB (Supplementary Table S4), and these associations still existed after partial correlation analyses adjusting for sex, age, BMI and eGFR (Supplementary Table S5). Third, compared with patients without hyperlipidemia (Supplementary Table S6), patients with hyperlipidemia had significantly higher TMAO levels (6.93 ± 5.42 μM vs. 4.23 ± 2.73 μM, *P* < 0.05), and the differences in other TMAO-related metabolites between the two patient groups were not significant (*P* > 0.05). Further analyses found that patients with hyper-TG or hypo-HDL-c had higher TMAO levels than patients without hyper-TG or hypo-HDL-c (7.94 ± 5.12 μM vs. 4.79 ± 4.00 μM or 6.97 ± 5.37 μM vs. 4.99 ± 3.95 μM, both *P* < 0.05), respectively. Patients with hyper-LDL-c had lower betaine levels than patients without hyper-LDL-c (33.17 ± 7.73 μM vs. 39.44 ± 10.61 μM, *P* < 0.05).

In univariate analyses (Supplementary Table S7), TMAO was positively correlated with TG and negatively correlated with HDL-c (*r* = 0.303 and − 0.405, respectively, both *P* < 0.05), and betaine was negatively correlated with LDL-c (*r* = − 0.308, *P* < 0.05). The correlations mentioned above still existed after partial correlation analyses adjusting for sex, age, BMI and eGFR (Table 2) or multivariate linear regression analyses adjusting for sex, age, BMI, eGFR and lipids TC, TG, LDL-c, HDL-c, ApoA1 and ApoB (Supplementary Tables S8 and S9, *P* < 0.05), and betaine was also significantly associated with TC after multivariate linear regression analyses (*P* < 0.05).

**Table 2:** Partial correlation analyses for the associations between TMAO-related metabolites and lipids before rosuvastatin therapy (*n* = 112)

Variables	TMAO		Choline		Carnitine		Betaine		GBB	
	*r*	*P*	*r*	*P*	*r*	*P*	*r*	*P*	*r*	*P*
TG	0.294	0.002	−0.020	0.839	−0.046	0.632	−0.008	0.936	−0.135	0.161
TC	0.156	0.106	0.093	0.338	0.073	0.451	−0.093	0.334	0.075	0.439
HDL-c	−0.316	0.001	−0.098	0.313	−0.009	0.923	−0.105	0.275	0.003	0.977
LDL-c	0.040	0.680	−0.037	0.702	−0.090	0.351	−0.269	0.005	0.043	0.654
ApoA1	−0.081	0.404	−0.075	0.441	0.070	0.468	−0.086	0.373	−0.035	0.715
ApoB	0.021	0.828	0.096	0.320	0.042	0.668	−0.028	0.772	0.076	0.429

*TMAO* Trimethylamine N-oxide, *TG* Triglycerides, *TC* Total cholesterol, *HDL-c* High-density lipoprotein cholesterol, *LDL-c* Low-density lipoprotein cholesterol, *ApoA1* Apolipoprotein A1, *ApoB* Apolipoprotein B, *GBB* γ-butyrobetaine. Partial correlation was performed by controlling for sex, age, body mass index and estimated glomerular filtration rate.

### Changes in TMAO-related metabolites after rosuvastatin therapy

Rosuvastatin therapy significantly decreased TG, TC, LDL-c and ApoB levels but increased HDL-c and ApoA1 levels (Supplementary Table S10, *P* < 0.05). Moreover, lower TMAO levels and higher carnitine, betaine and GBB levels were observed compared to baseline (3.82 ± 2.72 μM vs. 5.63 ± 4.52 μM, 83.23 ± 12.80 μM vs. 78.89 ± 15.93 μM, 44.67 ± 12.62 μM vs. 38.66 ± 10.47 μM and 0.11 ± 0.03 μM vs. 0.10 ± 0.02 μM, respectively, all *P* < 0.05). The changes in TMAO-related metabolites were still significant after adjustment for sex, age, BMI and eGFR by multivariate analyses (Table 3, *P* < 0.05). Compared with patients in the low-TMAO group (Supplementary Table S11), patients in the high-TMAO group had a greater reduction in TMAO levels after rosuvastatin therapy (3.85 ± 3.02 μM vs. 1.63 ± 1.50 μM, *P* < 0.05).

**Table 3:** Multivariate linear regression analyses for the changes in TMAO-related metabolites after rosuvastatin therapy (*n* = 112)

Variables	TMAO ^b^		Choline ^b^		Carnitine ^b^		Betaine ^b^		GBB ^b^	
	B	*P*	B	*P*	B	*P*	B	*P*	B	*P*
Sex	−0.198	0.618	− 0.939	0.080	−2.422	0.292	1.232	0.542	−0.012	0.024
Age	0.027	0.380	0.065	0.111	−0.122	0.488	−0.006	0.971	0.000	0.702
BMI	−0.010	0.854	0.007	0.923	−0.070	0.825	−0.072	0.802	0.001	0.437
eGFR	−0.037	0.276	0.059	0.188	−0.055	0.775	0.224	0.187	0.000	0.993
TMAO ^a^	0.386	< 0.001								
Choline ^a^			0.355	< 0.001						
Carnitine ^a^					0.371	< 0.001				
Betaine ^a^							0.756	< 0.001		
GBB ^a^									0.715	< 0.001

^a^indicates before rosuvastatin therapy

^b^indicates after rosuvastatin therapy

*TMAO* Trimethylamine N-oxide, *BMI* Body mass index, *eGFR* Estimated glomerular filtration rate, *GBB* γ-butyrobetaine. Variables including sex, age, BMI and eGFR were entered into the multivariate linear regression model.

In addition, (Supplementary Table S12), the correlation between TMAO and TG or between betaine and LDL-c disappeared after rosuvastatin therapy in univariate analysis (*r* = 0.050 and − 0.175, respectively, both *P* > 0.05), but correlations between TMAO and ApoB and between carnitine and LDL-c or ApoB were found to be significantly negative (*r* = − 0.241, − 0.296 and − 0.245, respectively, all *P* < 0.05). Further partial correlation analyses adjusting for sex, age, BMI and eGFR indicated that (Table 4), the negative correlation between carnitine and LDL-c or ApoB still existed (*P* < 0.05), and betaine was negatively correlated with TG (*P* < 0.05). After adjustment for the personal characteristics and lipids mentioned above, the negative association between carnitine and LDL-c or between betaine and TG was still significant (Supplementary Tables S13 and S14, *P* < 0.05), and a significantly positive association was also found between carnitine and TC (Supplementary Table S13, *P* < 0.05).

**Table 4:** Partial correlation analyses for the associations between TMAO-related metabolites and lipids after rosuvastatin therapy (*n* = 112)

Variables	TMAO		Choline		Carnitine		Betaine		GBB	
	*r*	*P*	*r*	*P*	*r*	*P*	*r*	*P*	*r*	*P*
TG	0.076	0.434	0.051	0.598	0.003	0.978	−0.202	0.035	−0.143	0.139
TC	−0.158	0.101	0.020	0.833	−0.104	0.281	−0.092	0.341	0.177	0.065
HDL-c	−0.172	0.074	0.046	0.633	0.052	0.591	0.088	0.361	0.063	0.513
LDL-c	−0.171	0.075	−0.071	0.460	−0.298	0.002	− 0.175	0.069	0.012	0.903
ApoA1	−0.059	0.539	0.035	0.716	−0.026	0.785	0.020	0.840	0.018	0.854
ApoB	−0.122	0.205	−0.075	0.440	−0.255	0.007	−0.150	0.119	−0.068	0.484

*TMAO* Trimethylamine N-oxide, *TG* Triglycerides, *TC* Total cholesterol, *HDL-c* High-density lipoprotein cholesterol, *LDL-c* Low-density lipoprotein cholesterol, *ApoA1* Apolipoprotein A1, *ApoB* Apolipoprotein B, *GBB* γ-butyrobetaine. Partial correlation was performed by controlling for sex, age, body mass index and estimated glomerular filtration rate.

## Discussion

In this study, we systematically investigated the associations between TMAO-related metabolites and blood lipids and assessed the potential impact of rosuvastatin therapy. We found that TMAO and betaine were significantly associated with TG, HDL-c, TC and/or LDL-c levels in univariate and/or multivariate analyses. Compared to baseline, lower TMAO levels and higher carnitine, betaine and GBB levels were observed after rosuvastatin therapy, while the lipids decreased significantly. Meanwhile, the associations between TMAO and TG and HDL-c or the association between betaine and LDL-c disappeared postrosuvastatin therapy. The associations between carnitine and TC and LDL-c and between betaine and TG were significant in multivariate linear regression analyses after rosuvastatin therapy, although there were no associations before rosuvastatin therapy.

Disorders of the gut microbiota and its function are associated with a series of chronic metabolic diseases, such as hyperlipidemia, ASCVD, DM and obesity [1, 4, 14, 21–25]. As important gut microbiota metabolites, TMAO-related metabolites have also been studied in recent years because of their proatherogenic effects. First, the levels of TMAO-related metabolites were influenced by diet habits, gut microbiota compositions and hepatic flavin-dependent monooxygenases and varied significantly in different individuals [26, 27]. Second, TMAO-related metabolite levels were also influenced by the physical conditions of the patients. Studies have indicated that TMAO is mainly excreted by the kidney, and impaired kidney function or reduced eGFR could decrease TMAO elimination [28, 29]. In addition, TMAO levels can increase with advancing age [30]. In this study, we found a similar phenomenon, as mentioned above. TMAO levels were positively correlated with patient age and BMI and negatively correlated with patient eGFR. In addition, we also found that betaine was negatively associated with BMI, and this result was also consistent with previous studies that found a negative correlation between betaine and BMI [17, 31]. In this study, we also assessed the associations among TMAO-related metabolites and found that TMAO was positively associated with choline and GBB, and most TMAO-related precursors were positively correlated with each other, except betaine and GBB. All the data mentioned above indicate that although choline, carnitine, betaine and GBB are called precursors of TMAO [32], there is no one-to-one relationship among them because of various influencing factors [27, 33].

In addition to the associations between TMAO-related metabolites and ASCVD, studies also found that changes in gut microbiota compositions were associated with variations in blood lipids [14, 15], and TMAO-related metabolites are involved in lipid metabolism [1, 2, 4]. Researchers found that TMAO could reduce the expression of the bile acid synthetic enzyme cytochrome P450 7A1 (CYP7A1), which is the key enzyme in cholesterol metabolism [2]. Mice administered a diet containing choline, carnitine or TMAO exhibited decreased reverse cholesterol transport [1, 2, 4]. A TMAO-containing diet could also reduce the expression of intestinal cholesterol transporters, which could excrete cholesterol through enterocytes [2]. All these data indicate that TMAO-related metabolites may affect cholesterol-containing lipid levels, and there may be some associations between TMAO-related metabolites and blood lipids. In this study, we found that patients with hyperlipidemia had significantly higher TMAO levels than patients without hyperlipidemia, but the differences in other TMAO-related metabolites between the two patient groups were not significant. Further analyses according to the types of hyperlipidemia indicated that patients with hyper-TG or hypo-HDL-c tended to have higher TMAO levels than patients without hyper-TG or hypo-HDL-c. Patients with hyper-LDL-c tended to have lower betaine levels than patients without hyper-LDL-c. The results of univariate correlation analyses also supported that TMAO had a positive correlation with TG and a negative correlation with HDL-c, and betaine had a negative correlation with LDL-c. Because of the significant correlations between TMAO-related metabolites and the personal characteristics or lipids mentioned above, we further analyzed the correlations between TMAO-related metabolites and lipids by partial correlation analyses and multivariate analyses and found that the associations mentioned above were still significant before rosuvastatin therapy. In addition, a positive association between betaine and TC was also found in multivariate analyses. We failed to find a negative association between betaine and TG, as previously reported [17], and the results of this study support an atherogenic role of TAMO [1, 8, 11]. However, the conflicting associations between betaine and LDL-c and TC make the negative association between betaine and ASCVD controversial [17, 31]. All these results indicate that TMAO-related metabolites are significantly associated with blood lipids, but these associations do not exist between every marker of TMAO-related metabolites and lipids. Although many studies have suggested significant associations between TMAO-related metabolites and ASCVD [1, 2, 4, 34, 35], their exact mechanisms are still unclear. Together with the laboratory findings about the influence of TMAO-related metabolites on inflammation and cholesterol metabolism [2, 4, 10, 11, 16], the findings of the associations between TMAO-related metabolites and lipids in our study may help to explain part of the associations between TMAO-related metabolites and ASCVD.

Previous studies found that many drugs could affect the composition of the gut microbiota [19, 36–38], but there is scarce information about their effect on the metabolites of the gut microbiota. Rosuvastatin is an excellent lipid-lowering drug that is used for ASCVD treatment and prevention and is commonly prescribed in developed and developing countries. Recent studies found that rosuvastatin could profoundly affect the gut microbiota composition [19, 38] and even the levels of TMAO-related metabolites [19]. The results of a small cohort study found that patients in the rosuvastatin therapy group had higher betaine and GBB levels than those in the placebo group, and the TMAO and carnitine levels in the rosuvastatin therapy group also tended to decrease and increase separately [19]. In this study, we found that blood lipids were significantly decreased after rosuvastatin therapy, and significantly lower TMAO levels and higher carnitine, betaine and GBB levels were also observed compared to baseline. These changes in TMAO-related metabolites still existed after multivariate analyses. The results of our study further support the findings of a previous study [19]. Although there was a lack of placebo controls in this study, the changed TMAO-related metabolites after rosuvastatin therapy were considered to be associated with the potential impact of rosuvastatin therapy according to the self-control study. Whether it is necessary and how to treat these elevated precursors is an ambiguous question. Therefore, more experimental studies are needed to clarify the potential mechanisms involved and identify potential therapeutic targets for use in clinical therapy.

Although previous studies found an influence of rosuvastatin therapy on gut microbiota and its metabolites [19, 38, 39], it is still unclear whether rosuvastatin could influence the bioenzymes associated with the metabolism of TMAO precursors directly. Therefore, it is difficult to explain the contradictory phenomenon of the decreased TMAO and its increased precursors after rosuvastatin therapy. In addition, although TMAO and lipids had a decreasing trend at the same time, it is also unclear whether there were some correlations between the reductions in TMAO and lipids. As previous studies reported that the compositions of gut microbiota were associated with the variations of lipids and could be influenced by statins [15, 19, 38, 39], the altered compositions of gut microbiota may be responsible for part of the lipid changes after rosuvastatin therapy. To some extent, the reduction in TMAO may be correlated with part of the reduction in lipids according to the altered gut microbiota after rosuvastatin therapy.

In addition, following the changes in TMAO-related metabolites and lipids after rosuvastatin therapy, the associations between them were also altered. The correlation between TMAO and TG or between betaine and LDL-c disappeared after rosuvastatin therapy, but significantly negative associations between carnitine and LDL-c and between betaine and TG emerged in univariate and/or multivariate analyses. Although we found a significant positive association between betaine and TC in multivariate analyses before rosuvastatin therapy, it disappeared after rosuvastatin therapy, and a positive association between carnitine and TC was found in multivariate analyses. Although studies have reported that carnitine is atherogenic [2, 7], the conflicting associations between carnitine and LDL-c and TC make the positive association between carnitine and ASCVD controversial. There is little information about the mechanisms of the changed associations after rosuvastatin therapy because of the unclear correlations between the changed TMAO-related metabolites and lipids discussed above, but the altered TMAO-related metabolites and lipid levels and the possible impact of rosuvastatin therapy on gut microbiota might be able to explain something. This may also be the reason for the inconsistency of some of our results with previous studies that did not consider the potential impact of statin therapy [17, 31]. The results of the study indicate that because of the wide use of statins, the potential impact of the changed TMAO-related metabolites should be considered when assessing their biological effect on ASCVD or their associations with ASCVD in the clinic.

### Comparisons with other studies and what the current study adds to the existing knowledge

As previous studies found that TMAO-related metabolites could influence cholesterol metabolism [2, 4, 16] and that the gut microbiota was associated with variations in lipids [14, 15], this study found that TMAO-related metabolites were also significantly associated with blood lipids, and these associations were changed but still existed after rosuvastatin therapy. In addition, similar to the results of a previous study [19], this study further demonstrated the potential impact of rosuvastatin therapy on TMAO-related metabolites.

### Study strengths and limitations

To the best of our knowledge, this is the first study to systematically explore the associations between TMAO-related metabolites and lipids and the impact of rosuvastatin therapy. There are also some limitations. First, enrolled patients with carotid plaque, suspected ASCVD and relatively old age inevitably create a certain amount of selection bias. Second, although we used a self-contrast method to evaluate the changes in TMAO-related metabolites before and after rosuvastatin therapy, it is still a weakness because of the lack of a placebo control. Third, although we tried to enlarge the sample size and our study represents the largest cohort to date evaluated for the associations between TMAO-related metabolites and blood lipids, it is still limited by the relatively small sample size. Fourth, although all the patients were given a recommended diet, instructed to avoid change and were even monitored weekly during the study, it was still difficult to avoid the potential influence of diet habits and a lack of reported specific diets. Finally, the usage of multiple drugs may inevitably lead to a potential impact on the gut microbiota and TMAO-related metabolites.

## Conclusions

This study suggests that TMAO-related metabolites are significantly associated with blood lipids, although some of them were changed after rosuvastatin therapy. Lower TMAO levels and higher betaine, carnitine and GBB levels were observed after rosuvastatin therapy compared to baseline. This study adds to our knowledge about the role of TMAO-related metabolites in ASCVD and indicates that although rosuvastatin therapy could decrease blood lipids and TMAO significantly, the elevated TMAO precursors postrosuvastatin therapy and their potential impact on ASCVD should be considered in the clinic.

## Supplementary Information

**Additional file 1: Figure S1.** The associations between the TMAO-related metabolites and personal characteristics (*n* = 112). **Table S1.** Baseline characteristics of patients without prior statin therapy (Group 1) and patients who received irregular statin therapy for less than one month (Group 2). **Table S2.** Baseline characteristics of males and females. **Table S3.** Correlations between the TMAO-related metabolites and personal characteristics (*n* = 112). **Table S4.** Correlations among the TMAO-related metabolites before statin therapy. **Table S5.** Partial correlation analyses for the associations among the TMAO-related metabolites before statin therapy. **Table S6.** Comparisons of the TMAO-related metabolites between different types of hyperlipidemia. **Table S7.** Correlations between the TMAO-related metabolites and blood lipids before rosuvastatin therapy (*n* = 112). **Table S8.** Multivariate linear regression analyses for the associations between TMAO and blood lipids before rosuvastatin therapy. **Table S9.** Multivariate linear regression analyses for the associations between betaine and blood lipids before rosuvastatin therapy. **Table S10.** The changes of TMAO-related metabolites after rosuvastatin therapy (*n* = 112). **Table S11.** The changes of TMAO-related metabolites between low-TMAO and high-TMAO groups after rosuvastatin therapy. **Table S12.** Correlations between the TMAO-related metabolites and blood lipids after rosuvastatin therapy (*n* = 112). **Table S13.** Multivariate linear regression analyses for the associations between carnitine and blood lipids after rosuvastatin therapy. **Table S14.** Multivariate linear regression analyses for the associations between betaine and blood lipids after rosuvastatin therapy.

## Data Availability

The data can be obtained from the corresponding author following the permission of the Ethics Committee of Beijing Tiantan Hospital.

## Abbreviations

ASCVD: Atherosclerotic cardiovascular disease
DM: Diabetes mellitus
TMAO: Trimethylamine N-oxide
GBB: γ-Butyrobetaine
MACE: Major adverse cardiovascular events
TG: Triglycerides
TC: Total cholesterol
HDL-c: High-density lipoprotein cholesterol
LDL-c: High-density lipoprotein cholesterol
ApoA1: Apolipoprotein A1
ApoB: Apolipoprotein B
BMI: Body mass index
eGFR: Estimated glomerular filtration rate
CYP7A1: Cytochrome P450 7A1
