# Supplementary material for: The associations between TMAO-related metabolites and blood lipids and the potential impact of rosuvastatin therapy
Source: Lipids Health Dis. 2022 Jul 21;21:60. doi: 10.1186/s12944-022-01673-3 (PMC9306211; doi:10.1186/s12944-022-01673-3)
Supplement: Supplementary file 1 — Additional file 1: Figure S1. The associations between the TMAO-related metabolites and personal characteristics (n = 112). Table S1. Baseline characteristics of patients without prior statin therapy (Group 1) and patients who received irregular statin therapy for less than one month (Group 2). Table S2. Baseline characteristics of males and females. Table S3. Correlations between the TMAO-related metabolites and personal characteristics (n = 112). Table S4. Correlations among the TMAO-related metabolites before statin therapy. Table S5. Partial correlation analyses for the associations among the TMAO-related metabolites before statin therapy. Table S6. Comparisons of the TMAO-related metabolites between different types of hyperlipidemia. Table S7. Correlations between the TMAO-related metabolites and blood lipids before rosuvastatin therapy (n = 112). Table S8. Multivariate linear regression analyses for the associations between TMAO and blood lipids before rosuvastatin therapy. Table S9. Multivariate linear regression analyses for the associations between betaine and blood lipids before rosuvastatin therapy. Table S10. The changes of TMAO-related metabolites after rosuvastatin therapy (n = 112). Table S11. The changes of TMAO-related metabolites between low-TMAO and high-TMAO groups after rosuvastatin therapy. Table S12. Correlations between the TMAO-related metabolites and blood lipids after rosuvastatin therapy (n = 112). Table S13. Multivariate linear regression analyses for the associations between carnitine and blood lipids after rosuvastatin therapy. Table S14. Multivariate linear regression analyses for the associations between betaine and blood lipids after rosuvastatin therapy. [file 12944_2022_1673_MOESM1_ESM.docx]

**Supplementary Figures and Tables**

**Figure S1** The associations between the TMAO-related metabolites and personal characteristics (n=112)


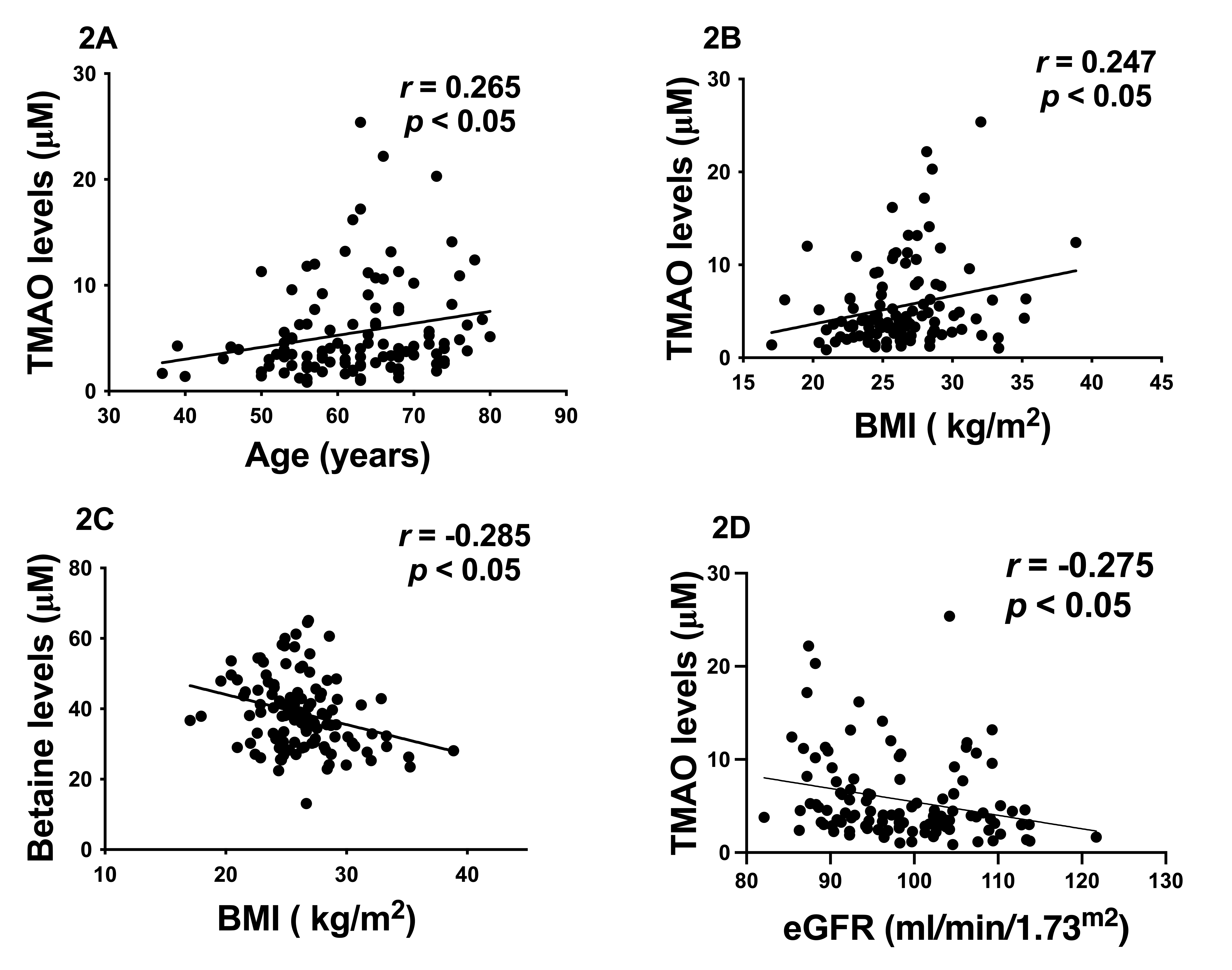


TMAO, trimethylamine N-oxide; BMI, body mass index; eGFR, estimated glomerular filtration rate.

**Table S1** Baseline characteristics of patients without prior statin therapy (Group 1) and patients who received irregular statin therapy for less than one month (Group 2)

| Variables | Group 1  (n=85) | Group 2  (n=27) | *P* |
| --- | --- | --- | --- |
| Male, n (%) | 51 (60) | 14 (51.85) | 0.455 |
| Age, years | 61.51±8.73 | 64.04±9.36 | 0.200 |
| BMI, kg/m^2^ | 26.24±3.66 | 26.16±3.03 | 0.911 |
| eGFR, mL/min/1.73^m2^ | 99.65±8.14 | 95.90±7.08 | 0.064 |
| Triglycerides, mmol/L | 1.94±0.96 | 1.94±1.02 | 0.980 |
| Total cholesterol, mmol/L | 4.34±1.00 | 4.14±0.95 | 0.348 |
| HDL-c, mmol/L | 1.21±0.40 | 1.18±0.34 | 0.692 |
| LDL-c, mmol/L | 2.62±0.74 | 2.56±0.74 | 0.724 |
| ApoA1, mmol/L | 1.25±0.19 | 1.29±0.17 | 0.331 |
| ApoB, mmol/L | 1.11±0.33 | 1.07±0.41 | 0.555 |
| TMAO, μM | 5.39±4.49 | 6.39±4.64 | 0.320 |
| Choline, μM | 13.30±3.13 | 13.11±2.77 | 0.784 |
| Carnitine, μM | 78.74±16.21 | 79.39±15.30 | 0.850 |
| Betaine, μM | 38.88±10.53 | 37.96±10.46 | 0.694 |
| GBB, μM | 0.10±0.02 | 0.09±0.03 | 0.741 |

Data are expressed as the mean ± standard deviation. BMI, body mass index; HDL-c, high-density lipoprotein cholesterol; LDL-c, low-density lipoprotein cholesterol; ApoA1, apolipoprotein A1; ApoB, apolipoprotein B; TMAO, trimethylamine N-oxide; GBB, γ-butyrobetaine.

**Table S2** Baseline characteristics of males and females

| Variables | Total patients  (n = 112) | Males  (n=65) | Females  (n=47) | *P* |
| --- | --- | --- | --- | --- |
| Age, years | 62.12±8.91 | 60.34±9.77 | 64.57±6.94 | **0.008** |
| BMI, kg/m^2^ | 26.22±3.50 | 25.87±3.26 | 26.71±3.79 | 0.215 |
| Smoke, n (%) | 55 (49.1) | 49 (75.4) | 6 (12.8) | **<0.001** |
| Alcohol, n (%) | 28 (25) | 27 (41.5) | 1 (2.1) | **<0.001** |
| Hypertension, n (%)  Anti-hypertension, n (%) | 85 (75.9)  82 (73.2) | 49 (75.4)  47 (72.3) | 36 (76.6)  35 (74.5) | 0.882  0.799 |
| Hyperlipidaemia, n (%)  Anti-hyperlipidaemia, n (%) | 58 (51.8)  27 (24.1) | 31 (47.7)  14 (21.5) | 27 (57.4)  13 (27.7) | 0.308  0.455 |
| CHD, n (%) | 94 (83.9) | 54 (83.1) | 40 (85.1) | 0.773 |
| eGFR, mL/min/1.73^m2^ | 98.74±8.03 | 99.99±7.89 | 97.01±7.97 | 0.052 |
| TG, mmol/L | 1.94±0.97 | 1.86±0.84 | 2.05±1.13 | 0.296 |
| TC, mmol/L | 4.30±1.01 | 4.19±0.96 | 4.45±1.06 | 0.171 |
| HDL-c, mmol/L | 1.21±0.39 | 1.18±0.39 | 1.24±0.39 | 0.407 |
| LDL-c, mmol/L | 2.55±0.88 | 2.59±0.73 | 2.62±0.76 | 0.847 |
| ApoA1, mmol/L | 1.26±0.18 | 1.25±0.18 | 1.28±0.19 | 0.310 |
| ApoB, mmol/L | 1.10±0.35 | 1.13±0.36 | 1.07±0.33 | 0.376 |
| TMAO, μM | 5.49±4.56 | 4.91±3.56 | 6.62±5.48 | 0.066 |
| Choline, μM | 13.25±3.03 | 13.46±2.70 | 12.97±3.45 | 0.406 |
| Carnitine, μM | 78.89±15.93 | 79.71±13.96 | 77.76±18.41 | 0.544 |
| Betaine, μM | 38.66±10.47 | 39.77±10.18 | 37.12±10.78 | 0.187 |
| GBB, μM | 0.10±0.02 | 0.10±0.02 | 0.09±0.02 | 0.099 |

Data are expressed as the mean ± standard deviation or n (%). BMI, body mass index; CHD, coronary heart disease; eGFR, estimated glomerular filtration rate; TG, triglycerides; TC, total cholesterol; HDL-c, high-density lipoprotein cholesterol; LDL-c, low-density lipoprotein cholesterol; ApoA1, apolipoprotein A1; ApoB, apolipoprotein B; TMAO, trimethylamine N-oxide; GBB, γ-butyrobetaine.

**Table S3** Correlations between the TMAO-related metabolites and personal characteristics (n = 112)

| Variables | Before statin therapy (n = 112) | | | | | | After statin therapy (n = 112) | | | | | |
| --- | --- | --- | --- | --- | --- | --- | --- | --- | --- | --- | --- | --- |
|  | Age | | BMI | | eGFR | | Age | | BMI | | eGFR | |
|  | *r* | *P* | *r* | *P* | *r* | *P* | *r* | *P* | *r* | *P* | *r* | *P* |
| TMAO | 0.265 | **0.005** | 0.247 | **0.009** | -0.275 | **0.003** | 0.260 | **0.006** | 0.144 | 0.131 | -0.320 | **0.001** |
| Choline | 0.116 | 0.223 | -0.079 | 0.407 | -0.108 | 0.256 | 0.090 | 0.344 | -0.042 | 0.663 | -0.002 | 0.986 |
| Carnitine | 0.086 | 0.366 | 0.001 | 0.989 | 0-.039 | 0.684 | -0.044 | 0.647 | -0.031 | 0.746 | 0.026 | 0.789 |
| Betaine | 0.141 | 0.137 | -0.285 | **0.002** | -0.095 | 0.318 | -0.005 | 0.956 | -0.201 | **0.033** | 0.066 | 0.487 |
| GBB | 0.064 | 0.503 | -0.071 | 0.456 | -0.082 | 0.389 | -0.045 | 0.637 | 0.002 | 0.980 | 0.009 | 0.928 |

TMAO, trimethylamine N-oxide; BMI, body mass index; eGFR, estimated glomerular filtration rate. GBB, γ-butyrobetaine.

**Table S4** Correlations among the TMAO-related metabolites before statin therapy

| Variables | TMAO | | Choline | | Carnitine | | Betaine | | GBB | |
| --- | --- | --- | --- | --- | --- | --- | --- | --- | --- | --- |
|  | *r* | *P* | *r* | *P* | *r* | *P* | *r* | *P* | *r* | *P* |
| TMAO |  |  | 0.250 | **0.008** | 0.155 | 0.102 | 0.009 | 0.922 | 0.221 | **0.019** |
| Choline | 0.250 | **0.008** |  |  | 0.394 | **<0.001** | 0.453 | **<0.001** | 0.514 | **<0.001** |
| Carnitine | 0.181 | 0.056 | 0.394 | **<0.001** |  |  | 0.307 | **<0.001** | 0.448 | **<0.001** |
| Betaine | 0.018 | 0.850 | 0.453 | **<0.001** | 0.315 | **0.001** |  |  | 0.116 | 0.223 |
| GBB | 0.217 | **0.022** | 0.422 | **<0.001** | 0.341 | **<0.001** | 0.116 | 0.223 |  |  |

TMAO, trimethylamine N-oxide; GBB, γ-butyrobetaine.

**Table S5** Partial correlation analyses for the associations among the TMAO-related metabolites before statin therapy

| Variables | TMAO | | Choline | | Carnitine | | Betaine | | GBB | |
| --- | --- | --- | --- | --- | --- | --- | --- | --- | --- | --- |
|  | *r* | *P* | *r* | *P* | *r* | *P* | *r* | *P* | *r* | *P* |
| TMAO |  |  | 0.206 | **0.032** | 0.080 | 0.412 | 0.021 | 0.826 | 0.228 | **0.018** |
| Choline | 0.206 | **0.032** |  |  | 0.388 | **<0.001** | 0.431 | **<0.001** | 0.387 | **<0.001** |
| Carnitine | 0.080 | 0.412 | 0.388 | **<0.001** |  |  | 0.314 | **0.001** | 0.294 | **0.002** |
| Betaine | 0.021 | 0.826 | 0.431 | **<0.001** | 0.314 | **0.001** |  |  | 0.160 | 0.097 |
| GBB | 0.228 | **0.018** | 0.387 | **<0.001** | 0.294 | **0.002** | 0.160 | 0.097 |  |  |

TMAO, trimethylamine N-oxide; GBB, γ-butyrobetaine. Partial correlation was used by controlling sex, age, body mass index and estimated glomerular filtration rate.

**Table S6** Comparisons of the TMAO-related metabolites between different types of hyperlipidemia

| Variables | Type of hyperlipidemia | | | | | | | | | |
| --- | --- | --- | --- | --- | --- | --- | --- | --- | --- | --- |
|  | Hyperlipidaemia | | TG ≥ 2.26 mmol/L | | TC ≥ 6.22 mmol/L | | LDL-c ≥ 4.14 mmol/L | | HDL-c ≤ 1.04 mmol/L | |
|  | No  (n = 54) | Yes  (n = 58) | No  (n = 82) | Yes  (n = 30) | No  (n = 103) | Yes  (n = 9) | No  (n = 98) | Yes  (n = 14) | No  (n = 76) | Yes  (n = 36) |
| TMAO (μM) | 4.23±2.73***** | 6.93±5.42 | 4.79±4.00***** | 7.94±5.12 | 5.54±4.23 | 6.65±7.41 | 5.50±4.25 | 6.56±6.26 | 4.99±3.95***** | 6.97±5.37 |
| Choline (μM) | 13.37±2.94 | 13.15±3.14 | 13.31±2.96 | 13.06±3.28 | 13.29±3.06 | 12.81±2.78 | 13.22±3.11 | 13.50±2.54 | 13.21±3.05 | 13.36±3.05 |
| Betaine (μM) | 39.47±9.9.53 | 37.90±11.31 | 39.29±10.33 | 36.95±10.84 | 39.11±10.53 | 33.48±8.61 | 39.44±10.61***** | 33.17±7.73 | 38.61±10.39 | 38.76±10.80 |
| Carnitine (μM) | 81.34±16.16 | 76.62±15.51 | 79.76±16.48 | 76.53±14.31 | 79.33±14.52 | 73.90±28.30 | 79.26±14.71 | 76.30±23.32 | 79.05±16.05 | 78.57±15.89 |
| GBB (μM) | 0.10±0.02 | 0.09±0.03 | 0.10±0.02 | 0.09±0.02 | 0.09±0.02 | 0.10±0.03 | 0.09±0.02 | 0.10±0.02 | 0.10±0.02 | 0.09±0.02 |

Data are expressed as the mean ± standard deviation. TMAO, trimethylamine N-oxide; TGs, triglycerides; TC, total cholesterol; HDL-c, high-density lipoprotein cholesterol; LDL-c, low-density lipoprotein cholesterol; GBB, γ-butyrobetaine. *Compared with “Yes” group, *P* < 0.05.

**Table S7** Correlations between the TMAO-related metabolites and blood lipids before rosuvastatin therapy (n = 112)

| Variables | TMAO | | Choline | | Carnitine | | Betaine | | GBB | |
| --- | --- | --- | --- | --- | --- | --- | --- | --- | --- | --- |
|  | *r* | *P* | *r* | *P* | *r* | *P* | *r* | *P* | *r* | *P* |
| TG | **0.303** | **0.001** | -0.025 | 0.792 | -0.052 | 0.589 | -0.060 | 0.531 | -0.138 | 0.146 |
| TC | 0.026 | 0.787 | 0.052 | 0.584 | 0.051 | 0.593 | -0.138 | 0.146 | 0.044 | 0.642 |
| HDL-c | **-0.405** | **<0.001** | -0.104 | 0.275 | -0.007 | 0.945 | -0.059 | 0.534 | 0.004 | 0.967 |
| LDL-c | -0.052 | 0.585 | -0.079 | 0.407 | -0.104 | 0.275 | **-0.308** | **0.001** | 0.000 | 0.999 |
| ApoA1 | -0.075 | 0.430 | -0.084 | 0.379 | 0.061 | 0.522 | -0.078 | 0.416 | -0.054 | 0.570 |
| ApoB | 000 | 0.998 | 0.102 | 0.284 | 0.044 | 0.646 | -0.006 | 0.947 | 0.080 | 0.403 |

TMAO, trimethylamine N-oxide; TG, triglycerides; TC, total cholesterol; HDL-c, high-density lipoprotein cholesterol; LDL-c, low-density lipoprotein cholesterol; ApoA1, apolipoprotein A1; ApoB, apolipoprotein B; GBB, γ-butyrobetaine.

**Table S8** Multivariate linear regression analyses for the associations between TMAO and blood lipids before rosuvastatin therapy

| Variables | B | 95% CI | *P* |
| --- | --- | --- | --- |
| Sex | 1.048 | -0.58-2.68 | 0.205 |
| Age | 0.095 | -0.03-0.22 | 0.135 |
| BMI | 0.181 | -0.04-0.41 | 0.112 |
| eGFR | -0.015 | -0.15-0.12 | 0.834 |
| TG | 0.858 | 0.02-2.70 | **0.046** |
| TC | 0.948 | -0.32-2.22 | 0.141 |
| HDL-c | -3.062 | -5.36 - -0.76 | **0.009** |
| LDL-c | -0.830 | -2.48-0.83 | 0.322 |
| ApoA1 | -0.507 | -5.31–4.29 | 0.834 |
| ApoB | 0.339 | -1.93–2.60 | 0.767 |

TMAO, trimethylamine N-oxide; CI, confidence interval; BMI, body mass index; eGFR, estimated glomerular filtration rate; TG, triglycerides; TC, total cholesterol; HDL-c, high-density lipoprotein cholesterol; LDL-c, low-density lipoprotein cholesterol; ApoA1, apolipoprotein A1; ApoB, apolipoprotein B. Variables, including sex, age, BMI, eGFR, TG, TC, HDL-c, LDL-c, ApoA1 and ApoB, were entered into the multivariate linear regression model.

**Table S9** Multivariate linear regression analyses for the associations between betaine and blood lipids before rosuvastatin therapy

| Variables | B | 95% CI | *P* |
| --- | --- | --- | --- |
| Sex | -2.472 | -6.40-1.46 | 0.215 |
| Age | 0.159 | -0.14-0.46 | 0.297 |
| BMI | -0.776 | -1.32 - -0.24 | **0.005** |
| eGFR | 0.030 | -0.31-0.36 | 0.861 |
| TG | -0.403 | -2.43-1.63 | 0.695 |
| TC | 3.174 | 0.12-6.23 | **0.042** |
| HDL-c | -1.443 | -6.98-4.10 | 0.607 |
| LDL-c | -6.826 | -10.82 - -2.84 | **0.001** |
| ApoA1 | -5.287 | -16.86–6.29 | 0.367 |
| ApoB1 | 0.996 | -4.47–6.46 | 0.718 |

CI, confidence interval; BMI, body mass index; eGFR, estimated glomerular filtration rate; TG, triglycerides; TC, total cholesterol; HDL-c, high-density lipoprotein cholesterol; LDL-c, low-density lipoprotein cholesterol; ApoA1, apolipoprotein A1; ApoB, apolipoprotein B. Variables, including sex, age, BMI, eGFR, TG, TC, HDL-c, LDL-c, ApoA1 and ApoB, were entered into the multivariate linear regression model.

**Table S10** The changes of TMAO-related metabolites after rosuvastatin therapy (n = 112)

| Variables | Rosuvastatin therapy | | *P* |
| --- | --- | --- | --- |
|  | Before | After |  |
| TG, mmol/L | 1.94±0.97 | 1.43±0.83 | <0.001 |
| TC, mmol/L | 4.30±0.99 | 3.54±0.87 | <0.001 |
| HDL-c, mmol/L | 1.21±0.39 | 1.39±0.42 | <0.001 |
| LDL-c, mmol/L | 2.60±0.74 | 1.99±0.62 | <0.001 |
| ApoA1, mmol/L | 1.26±0.18 | 1.43±0.29 | <0.001 |
| ApoB, mmol/L | 1.10±0.35 | 0.89±0.23 | <0.001 |
| TMAO, μM | 5.63±4.52 | 3.82±2.72 | <0.001 |
| Choline, μM | 13.51±3.03 | 13.03±2.89 | 0.470 |
| Carnitine, μM | 78.89±15.93 | 83.23±12.80 | 0.003 |
| Betaine, μM | 38.66±10.47 | 44.67±12.62 | <0.001 |
| GBB, μM | 0.10±0.02 | 0.11±0.03 | <0.001 |

Data are expressed as the mean ± standard deviation. TMAO, trimethylamine N-oxide; TG, triglycerides; TC, total cholesterol; HDL-c, high-density lipoprotein cholesterol; LDL-c, low-density lipoprotein cholesterol; ApoA1, apolipoprotein A1; ApoB, apolipoprotein B; GBB, γ-butyrobetaine.

**Table S11** The changes of TMAO-related metabolites between low-TMAO and high-TMAO groups after rosuvastatin therapy

| Variables | TMAO ≤ 3.92μM (n = 56) | | | TMAO > 3.92μM (n = 56) | | |
| --- | --- | --- | --- | --- | --- | --- |
|  | Rosuvastatin therapy | | | Rosuvastatin therapy | | |
|  | Before1 | After1 | D1 | Before2 | After2 | D2 |
| TMAO, μM | 2.83±1.34 | 2.75±2.01 | 1.63±1.50 | 8.43±4.85 | 4.88±2.93**^&^** | 3.85±3.02* |
| Choline, μM | 12.50±2.81 | 12.88±2.82 | 2.37±1.65 | 14.01±3.09 | 13.18±2.97 | 2.63±2.53 |
| Carnitine, μM | 75.21±15.69 | 79.97±11.00**^#^** | 12.71±7.37 | 82.58±15.44 | 86.48±13.71 | 13.96±9.18 |
| Betaine, μM | 39.26±10.90 | 43.57±12.30**^#^** | 8.76±6.99 | 38.06±10.09 | 45.78±12.95**^&^** | 10.95±6.46 |
| GBB, μM | 0.09±0.02 | 0.10±0.03**^#^** | 0.02±0.01 | 0.10±0.02 | 0.11±0.03**^&^** | 0.03±0.02 |

TMAO, trimethylamine N-oxide; GBB, γ-butyrobetaine; D, the difference of TMAO-related metabolites before and after rosuvastatin therapy. **^#^**Compared to group before1, *p* < 0.05. **^&^**Compared to group before2, *p* < 0.05. *Compared to group D1, *p* < 0.05.

**Table S12** Correlations between the TMAO-related metabolites and blood lipids after rosuvastatin therapy (n = 112)

| Variables | TMAO | | Choline | | Carnitine | | Betaine | | GBB | |
| --- | --- | --- | --- | --- | --- | --- | --- | --- | --- | --- |
|  | *r* | *P* | *r* | *P* | *r* | *P* | *r* | *P* | *r* | *P* |
| TG | 0.050 | 0.599 | 0.082 | 0.392 | -0.018 | 0.854 | -0.124 | 0.193 | -0.163 | 0.086 |
| TC | -0.181 | 0.057 | 0.025 | 0.796 | -0.098 | 0.304 | -0.061 | 0.523 | 0.174 | 0.066 |
| HDL-c | **-0.253** | **0.007** | -0.039 | 0.686 | 0.063 | 0.510 | 0.138 | 0.145 | 0.050 | 0.604 |
| LDL-c | -0.172 | 0.069 | -0.074 | 0.436 | **-0.296** | **0.002** | -0.175 | 0.065 | 0.011 | 0.908 |
| ApoA1 | -0.025 | 0.791 | 0.051 | 0.592 | -0.023 | 0.810 | 0.058 | 0.544 | 0.007 | 0.940 |
| ApoB | -0.241 | 0.011 | -0.089 | 0.350 | **-0.245** | **0.009** | -0.154 | 0.104 | -0.063 | 0.507 |

TMAO, trimethylamine N-oxide; TG, triglycerides; TC, total cholesterol; HDL-c, high-density lipoprotein cholesterol; LDL-c, low-density lipoprotein cholesterol; ApoA1, apolipoprotein A1; ApoB, apolipoprotein B; GBB, γ-butyrobetaine.

**Table S13** Multivariate linear regression analyses for the associations between carnitine and blood lipids after rosuvastatin therapy

| Variables | B | 95% CI | *P* |
| --- | --- | --- | --- |
| Sex | -3.141 | -8.15–1.87 | 0.217 |
| Age | -0.073 | -0.45–0.30 | 0.702 |
| BMI | 0.183 | -0.51–0.88 | 0.602 |
| eGFR | -0.016 | -0.44–0.41 | 0.941 |
| TG | 0.243 | -3.22–2.73 | 0.872 |
| TC | 5.215 | 0.51–9.92 | **0.030** |
| HDL-c | 2.304 | -4.24–8.85 | 0.487 |
| LDL-c | -10.518 | -17.20 – -3.83 | **0.002** |
| ApoA1 | -5.901 | -16.13–4.33 | 0.255 |
| ApoB | -5.493 | -17.27-6.28 | 0.357 |

TMAO, trimethylamine N-oxide; CI, confidence interval; BMI, body mass index; eGFR, estimated glomerular filtration rate; TG, triglycerides; TC, total cholesterol; HDL-c, high-density lipoprotein cholesterol; LDL-c, low-density lipoprotein cholesterol; ApoA1, apolipoprotein A1; ApoB, apolipoprotein B. Variables, including sex, age, BMI, eGFR, TGs, TC, HDL-c, LDL-c, ApoA1 and ApoB, were entered into the multivariate linear regression model.

**Table S14** Multivariate linear regression analyses for the associations between betaine and blood lipids after rosuvastatin therapy

| Variables | B | 95% CI | *P* |
| --- | --- | --- | --- |
| Sex | -1.288 | -6.33–3.76 | 0.614 |
| Age | 0.067 | -0.31–0.45 | 0.728 |
| BMI | -0.525 | -1.22–0.17 | 0.140 |
| eGFR | 0.153 | -0.28–0.58 | 0.482 |
| TG | -3.233 | -6.23– -0.24 | **0.035** |
| TC | 2.324 | -2.41–7.06 | 0.333 |
| HDL-c | 1.325 | -5.27–7.91 | 0.691 |
| LDL-c | -5.259 | -11.99–1.47 | 0.124 |
| ApoA1 | -0.177 | -10.48–10.13 | 0.973 |
| ApoB | -2.662 | -14.52–9.19 | 0.657 |

TMAO, trimethylamine N-oxide; CI, confidence interval; BMI, body mass index; eGFR, estimated glomerular filtration rate; TG, triglycerides; TC, total cholesterol; HDL-c, high-density lipoprotein cholesterol; LDL-c, low-density lipoprotein cholesterol; ApoA1, apolipoprotein A1; ApoB, apolipoprotein B. Variables, including sex, age, BMI, eGFR, TG, TC, HDL-c, LDL-c, ApoA1 and ApoB, were entered into the multivariate linear regression model.
